# Depletion of Neutrophils Exacerbates the Early Inflammatory Immune Response in Lungs of Mice Infected with *Paracoccidioides brasiliensis*

**DOI:** 10.1155/2016/3183285

**Published:** 2016-08-25

**Authors:** Paula Andrea Pino-Tamayo, Juan David Puerta-Arias, Damaris Lopera, Martha Eugenia Urán-Jiménez, Ángel González

**Affiliations:** ^1^Medical and Experimental Mycology Unit, Corporación para Investigaciones Biológicas (CIB), Medellín, Colombia; ^2^Basic and Applied Microbiology Research Group (MICROBA), School of Microbiology, Universidad de Antioquia, Calle 70 No. 52-21, Medellín, Colombia

## Abstract

Neutrophils predominate during the acute phase of the* Paracoccidioides brasiliensis* infection. Herein, we determined the role of the neutrophil during the early stages of experimental pulmonary paracoccidioidomycosis using a monoclonal antibody (mAb) specific for neutrophils. Male BALB/c mice were inoculated intranasally with 1.5 × 10^6^ or 2 × 10^6^ *P. brasiliensis* yeast cells. The mAb was administered 24 h before infection, followed by doses every 48 h until mice were sacrificed. Survival time was evaluated and mice were sacrificed at 48 h and 96 h after inoculation to assess cellularity, fungal load, cytokine/chemokine levels, and histopathological analysis. Neutrophils from mAb-treated mice were efficiently depleted (99.04%). Eighty percent of the mice treated with the mAb and infected with 1.5 × 10^6^ yeast cells died during the first two weeks after infection. When mice were treated and infected with 2 × 10^6^ yeast cells, 100% of them succumbed by the first week after infection. During the acute inflammatory response significant increases in numbers of eosinophils, fungal load and levels of proinflammatory cytokines/chemokines were observed in the mAb-treated mice. We also confirmed that neutrophils are an important source of IFN-*γ* and IL-17. These results indicate that neutrophils are essential for protection as well as being important for regulating the early inflammatory immune response in experimental pulmonary paracoccidioidomycosis.

## 1. Introduction

Neutrophils have been considered to be crucial players in immune defense against bacterial and fungal pathogens. They participate as the first line of innate immunity and as effectors of adaptive immunity, contributing to the development of inflammatory reactions [1, 2]. These cells exert mechanisms of defense, such as phagocytosis [3, 4], intracellular, and extracellular pathogen destruction by chemical means [4, 5], neutrophil extracellular traps (NETs) formation [4, 6], and the production of numerous cytokines, chemokines, and growth factors [7, 8] used to defeat microbial pathogens [4, 9]. Furthermore, recent discoveries have suggested novel roles of neutrophils in a variety of immunological and inflammatory aspects such as immunity against intracellular pathogens (viruses and intracellular bacteria), shaping of adaptive immunity at different levels, and new potential roles for NETs beyond antimicrobial functions [2].

Paracoccidioidomycosis (PCM), caused by the dimorphic pathogenic fungus of the genus* Paracoccidioides*, is considered to be one of the most frequent systemic mycoses in Latin America. It is estimated that 10 million people are infected with the fungal agent although only about 1-2% develop mycosis [10, 11]. Some studies based on animal models have suggested that neutrophils play a significant role during the infection, specifically during the acute inflammatory phase [24–96 h after infection], representing more than 85% of inflammatory cells in the infected tissue [12, 13]. It has recently been suggested that, during infection with* P. brasiliensis*, human neutrophils may modulate the adaptive immune system toward a protective response mediated by a T helper 1 (Th1) pattern via Toll-Like Receptor 4 (TLR4) and Dectin-1, inducing interferon- (IFN-) *γ* production upon stimulation with the combination of interleukin- (IL-) 12 and IL-15 [14]. Another study suggested a similar modulating effect of neutrophils on the innate and adaptive immune response against* P. brasiliensis* through the production of cytokines and lipid mediators, which may lead to a pro- or anti-inflammatory pattern depending on the fungal strain and the pattern-recognition receptors (PRRs) associated with its recognition by these phagocytic cells [15].

Many strategies have been used to delineate neutrophil functions in different animal models of disease [2, 16–18]. Those strategies include antibody- (Ab-) mediated neutrophil depletion by anti-Gr1 or anti-Ly6G Abs, with the latter being more specific for these phagocytic cells [19], or genetic studies inducing the deletion of the neutrophil lineage [20, 21]. Depletion of these cells using mAbs has also been used to understand how neutrophils work in fungal infections [22–25]. Neutrophil depletion using anti-Gr1 Abs in two different mouse models of PCM indicates that the host genetic pattern has a significant influence on the immunoprotective and immunoregulatory functions of neutrophils; thus, resistant (A/J) or susceptible (B10.A) mice to* P. brasiliensis* infection were depleted of neutrophils and evaluated one week after infection and mainly during the adaptive immune response development to this fungal infection [26]. In this investigation, the authors observed that the mean survival time of neutrophil-depleted susceptible mice was 22.5 weeks in comparison with resistant mice that showed a mean survival time of 34.2 weeks; in addition, higher levels of IL-4, IL-12, and antibodies associated with Th1 and Th2 profiles in the susceptible strain were observed. By contrast, increased IL-12, granulocyte-macrophage colony-stimulating factor (GM-CSF), and Th1-regulated antibodies were found in the neutrophil-depleted resistant mice [26], without any alteration of delayed-type hypersensitivity reactions.

Taking into account that neutrophils are crucial cells during the first hours to encounter with the pathogen and they play an important role in immunopathology of fungal diseases, we aimed to evaluate the participation of these phagocytic cells during the early development of the immune response in the experimental PCM using an intermediate susceptible animal model to this infection. Herein, we observed that neutrophil-depleted animals succumbed during the first two weeks of* P. brasiliensis* infection accompanied by an increase in numbers of eosinophils and fungal load as well as proinflammatory cytokine and chemokines. In addition, we confirmed that these phagocytic cells are an important source of IFN-*γ* and IL-17.

## 2. Materials and Methods

### 2.1. Animals

BALB/c mice were obtained from the breeding colony maintained at Corporación para Investigaciones Biológicas (CIB) (Medellín, Colombia). Eight-week-old male mice were used to conduct all procedures. Mice were separated into two experimental groups consisting of infected or noninfected control mice. Mice in both groups were allocated to the following treatment regimens for each evaluation time: untreated, isotype control-treated, and neutrophil-depleted ones. Animal experiments were approved by the Institutional Ethics Committee and followed Colombian (Law 84/1989, Resolution numberNo. 8430/1993), European Union, and Canadian Council on Animal Care regulations.

### 2.2. Fungus

A highly virulent isolate of* P. brasiliensis* (Pb18) was used throughout this study [27]. Pb18 yeast cells were subcultured weekly on slant agar tubes with Sabouraud Dextrose Agar (Difco Laboratories, Detroit, MI, USA) supplemented with 0.14% L-asparagine (Sigma-Aldrich, Saint Louis, MO, USA), 0.01% thiamine hydrochloride (Sigma-Aldrich, Saint Louis, MO, USA), and 100 U/milliliter (mL) Penicillin-100 *μ*g/mL Streptomycin (GIBCO Invitrogen Corporation, Carlsbad, CA, USA) and incubated at 36°C in 5% CO_2_. Yeast cells were subsequently transferred into 100 mL Sabouraud Dextrose Broth (Difco Laboratories, Detroit, MI, USA), supplemented with L-asparagine, thiamine hydrochloride, and antibiotics solution as described above and incubated during 4 days at 36°C, rotating at 150 revolutions per minute (rpm). After this period, the yeast suspension was pelleted by centrifugation at 1,400 ×g, 10°C for 10 minutes, washed once using 1x phosphate-buffered saline (PBS), pH = 7.4 (GIBCO Invitrogen Corporation, Carlsbad, CA, USA), and centrifuged as described above; the pellet was then resuspended in 30 mL 1x PBS and passed 20 times through a 21G × 1.5-inch needle using a 10 mL syringe in order to obtain 65–80% individual yeast cells. The yeast suspension was allowed to settle for 10 minutes and 25 mL supernatant (with individual yeast cells) was transferred to a new tube, centrifuged, resuspended, and counted in a hemocytometer to determine the percentage of individual yeast cells and their viability using Janus Green vital dye (Acros Organics, Geel, Belgium). The number of yeast cells was adjusted to 1.5 × 10^6^ or 2 × 10^6^ contained in 60 *μ*L in order to infect mice.

### 2.3. *Paracoccidioides brasiliensis* Infection

Intranasal inoculation of 1.5 × 10^6^ or 2 × 10^6^ yeast cells contained in 60 *μ*L PBS was performed in mice after the animals had been anesthetized by intramuscular injection of 50 *μ*L Ketamine-Xylazine solution at 80 mg/kg Ketamine (Laboratorios Biosano, Santiago, Chile) and 8 mg/kg Xylazine (Bayer S.A., Bogotá, Colombia). The 60 *μ*L total inoculum was split into two doses of 30 *μ*L each and instilled intranasally within a 5–10-minute period. Noninfected (control) mice were inoculated with 60 *μ*L of 1x PBS.

### 2.4. Depletion of Neutrophils

Mice were injected intraperitoneally with 200 *μ*g of monoclonal Ab (mAb) anti-Ly6G, clone 1A8 (Bio X Cell, West Lebanon, NH, USA) contained in 200 *μ*L PBS. Control mice were injected with an equivalent amount of an isotype control Immunoglobulin (Ig) G2a, clone 2A3 (Bio X Cell, West Lebanon, NH, USA). In order to evaluate the acute phase of* P. brasiliensis* infection, mAb treatment was administered 24 h before inoculation (with PBS or* P. brasiliensis*) followed by doses every 48 h until mice were sacrificed (48 h or 96 h postinfection).

### 2.5. Survival Curve

Survival of BALB/c mice infected with 1.5 × 10^6^ or 2 × 10^6^ yeast cells of* P. brasiliensis* and exposed to every treatment during the acute phase of the infection was verified daily for a period of 12 weeks; thus, mAb treatment was administered 24 h before infection followed by additional doses every 48 h over a period of two weeks. Two survival curves were obtained with the corresponding replicates. Survival data were analyzed by Kaplan-Meier curves using the Log-rank test.

### 2.6. CFU Determination

During the acute phase of infection untreated, isotype control-treated and anti-Ly6G treated mice were sacrificed at 48 h and 96 h after infection. Lungs were removed, weighed, and homogenized in 2 mL sterile 1x PBS/Penicillin-Streptomycin solution [(GIBCO Invitrogen Corporation, Carlsbad, CA, USA) to a final concentration of 100 U/mL Penicillin-100 *μ*g/mL Streptomycin] using a gentle MACS Dissociator (Miltenyi Biotec, Teterow, Germany). Homogeneous suspensions were diluted (1 : 100, 1 : 1,000, and 1 : 10,000) and 0.5 mL of each dilution was plated on Petri dishes with Brain Heart Infusion (BHI) agar medium (BD BBL, Franklin Lakes, NJ, USA) supplemented with D-(+)-Glucose [(Sigma-Aldrich, Saint Louis, MO, USA) to a final concentration of 0.5%], horse serum [(GIBCO Invitrogen Corporation, Carlsbad, CA, USA) to a final concentration of 4%, previously inactivated by heating at 56°C for 30 min], and EDTA [(Sigma-Aldrich, Saint Louis, MO, USA) to a final concentration of 300 *μ*M], followed by incubation at 36°C, 5% CO_2_. Colony-forming units (CFU) counts were assessed 11 days after cultivation. The following formula was applied: CFU/gram (g) of tissue = total number of colonies multiplied by the total dilution factor. The total dilution factor was obtained by multiplying dilution factors for each of the following parameters: homogenized tissue (total volume of homogenized tissue/g of tissue), volume plated on Petri dishes (0.5 mL), and diluted homogeneous suspension plated on Petri dishes. The data was then transformed into log_10_ CFU/g of tissue.

### 2.7. Cytokine and Chemokine Measurement

Groups of mice were sacrificed as mentioned before at 48 h and 96 h after inoculation with PBS or* P. brasiliensis*. Lungs were removed and homogenized as previously described. The homogeneous suspension was transferred into microcentrifuge tubes and a protease inhibitor cocktail (Roche Applied Science, Mannheim, Germany) was added, mixed, and centrifuged at 3,000 rpm, 4°C for 5 minutes. Supernatants were aliquoted and stored at −70°C until their use. Cytokines and chemokines [CC chemokine ligand 11 (CCL11) (Eotaxin), granulocyte colony-stimulating factor (G-CSF), GM-CSF, IFN-*γ*, IL-1*α*, IL-1*β*, IL-2, IL-3, IL-4, IL-5, IL-6, IL-7, IL-9, IL-10, IL-12(p40), IL-12(p70), IL-13, IL-15, IL-17, CXC chemokine ligand 10 (CXCL10) (interferon-inducible protein-10 (IP-10)), CXCL1 (keratinocyte chemoattractant (KC)), leukemia inhibitory factor (LIF), CXCL5 (lipopolysaccharide induced CXC chemokine (LIX)), CCL2 (monocyte chemoattractant protein 1 (MCP-1)), macrophage colony-stimulating factor (M-CSF), CXCL9 (monokine induced by interferon-gamma (MIG)), CCL3 (macrophage-inflammatory protein 1*α* (MIP-1*α*)), CCL4 (MIP-1*β*), CXCL2 (MIP-2), CCL5 (regulated upon activation normal, RANTES), tumor necrosis factor- (TNF-) *α*, and vascular endothelial growth factor (VEGF)] [28, 29] were determined from those supernatants by a multiplex assay using a commercial kit and the Luminex 200 system (EMD Millipore, Billerica, MA, USA).

### 2.8. Flow Cytometry

Groups of animals were sacrificed as previously described at the time intervals detailed above. Lungs of mice were removed and homogenized using 40 and 70 *μ*m sterile cell strainers (Thermo Fisher Scientific Inc., Waltham, MA, USA) in RPMI plus 1% FBS [fetal bovine serum (Sigma-Aldrich, Saint Louis, MO, USA) previously inactivated by heating to 56°C for 30 minutes]. The cell suspension was pelleted by centrifugation at 1,500 rpm, 10°C for 10 minutes, and red blood cells lysed using 3 mL of ACK Lysing Buffer (GIBCO Invitrogen Corporation, Carlsbad, CA, USA) for 5 minutes at room temperature. Six mL of RPMI plus 1% FBS was then added to stop the reaction and wash the cells. After centrifugation, the pellet was resuspended in 1 mL of RPMI plus 10% FBS and cells were counted in a hemocytometer. The cell suspension was split into several wells on a 96-well plate in order to determine the following cell populations: neutrophils, eosinophils, dendritic cells (DCs), macrophages, B cells, CD8 T cells, CD4 T cells, and natural killer cells (NK cells). Fc receptors were blocked using a purified rat anti-mouse CD16/CD32 (BD Pharmingen, San Diego, CA, USA) and cells were immunostained in fluorescence-activated cell sorter (FACS) buffer (1x PBS/0.5% FBS) using fluorescent mAbs against murine surface molecules and isotype controls as follows: fluorescein isothiocyanate- (FITC-) rat IgG_2b_ *κ*, phycoerythrin- (PE-) rat IgG_2a_ *κ*, allophycocyanin- (APC-) rat IgG_1_ *κ*, FITC-anti-CD45, APC-anti-Ly-6G, APC-Cy7-anti-Ly-6G and APC-Cy7-anti-Ly-6C, PE-anti-CD11b, APC-anti-CD11c, PE-anti-Siglec-F, PE-anti-Mac-3, PE-anti-CD23, APC-anti-IgM, PE-anti-CD3 molecular complex, peridinin chlorophyll protein- (PerCP-) anti-CD8a, PerCP-Cy5.5-anti-CD4, APC-anti-NK-1.1, PE-Cy7-anti-IFN-*γ*, and APC-Cy7-anti-IL-17A (BD Pharmingen, San Diego, CA, USA). For intracellular analysis of IFN-*γ* and IL-17, cells were treated with Cytofix/Cytoperm*™* solution (BD Biosciences, San Diego, CA, USA) and Perm/Wash*™* solution (BD Biosciences, San Diego, CA, USA) before intracellular staining. After incubation with the specific mAb, cell suspensions were washed once in FACS buffer and fixed with FACS buffer/1% PFA (Paraformaldehyde, Carlo Erba, Barcelona, Spain). Cells were analyzed using a FACS Canto II system (BD Biosciences, San Jose, CA, USA) and FlowJo V10 software (FlowJo, LLC, Data Analysis Software, Ashland, OR, USA). Cell populations were analyzed as follows: (a) cell events in region 1 (R1) were gated by forward scatter versus side scatter areas to exclude cell debris; (b) CD45^+^ events were then gated from R1 by side scatter area versus CD45 staining to establish the R2 region, from which (c) cell events were gated according to the specific surface marker in order to determine the specific cell subpopulation. To determine intracellular cytokines, neutrophils were analyzed as follows: (a) cell events in R1 were gated by forward scatter versus side scatter areas; (b) CD45^+^ events were gated from R1 by side scatter area versus CD45 staining to establish the R2 region; (c) cell events were gated from R2 according to the specific surface markers for neutrophils establishing the R3 region, from which (d) cell events were gated according to the specific surface markers for neutrophils versus IFN-*γ* staining or neutrophils versus IL-17A staining. The total number of leukocytes was calculated by multiplying the total number of cells (counted in a hemocytometer) by the percentage of CD45^+^ cells, as determined by flow cytometry. The number of each leukocyte subpopulation (neutrophils: CD45^+^/CD11b^+^/Gr1^+^, eosinophils: CD45^+^/CD11c^−^/SiglecF^+^, alveolar macrophages: CD45^+^/CD11c^+^/CD11b^low/med^, DCs: CD45^+^/CD11c^+^/CD11b^high^, tissue macrophages: CD45^+^/CD11c^+^/Mac3^+^, B cells: CD45^+^/CD23^+^/IgM^+^, CD8 T cells: CD45^+^/CD3^+^/CD8^+^, CD4 T cells: CD45^+^/CD3^+^/CD4^+^, and NK cells: CD45^+^/CD3^−^/NK1.1^+^) was determined by multiplying the percentage of each gated subpopulation by the total number of leukocytes (CD45^+^ population).

### 2.9. Histopathological Analysis

Mice were sacrificed as previously described at the time intervals detailed above. Before their removal, lungs were perfused by injecting 10 mL of 1x PBS to wash out red blood cells from the tissue, after which 10 mL of formalin [formaldehyde solution (EM Science, Gibbstown, NJ, USA) to a final concentration of 4%, sodium dihydrogen phosphate (Merck, Darmstadt, Germany) to a final concentration of 0.15 M, and sodium hydroxide (Sigma-Aldrich, Saint Louis, MO, USA) to a final concentration of 0.11 M] [30] was used to fix tissues. Lungs were removed and submerged in 4% formalin until being processed. Fixed tissues were embedded in paraffin, cut, and stained using hematoxylin and eosin (H&E) to determine the lung inflammatory response and methenamine silver to identify* P. brasiliensis* yeast cells. Tissue slices were sent to a Pathology Laboratory for analysis.

### 2.10. Data Analysis

The data analysis was performed using Graph Pad Prism software version 5 (GraphPad Software Inc., La Jolla, CA, USA). Fungal load, flow cytometry, cytokines, and chemokines levels were expressed as medians and interquartile range (IQR). The Mann-Whitney test was used for comparisons between groups. Survival data were analyzed by Kaplan-Meier curves using the Log-rank test. Values of *P* < 0.05 were considered to be significant.

## 3. Results

### 3.1. Neutrophils Were Efficiently Depleted Using the Specific mAb Anti-Ly6G

**Figure 1:**
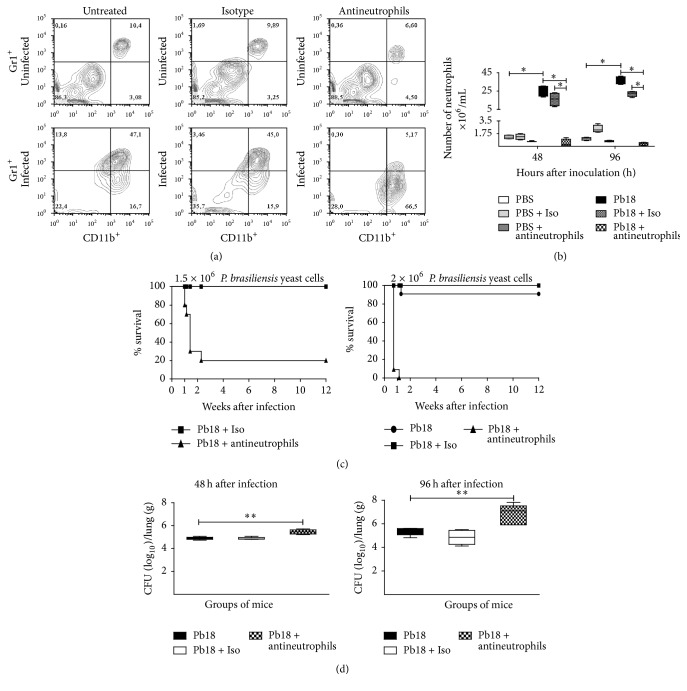
The anti-Ly6G mAb efficiently depleted neutrophils demonstrating their essential role during the early stages of* P. brasiliensis* infection. BALB/c mice were intranasally inoculated with PBS or 1.5 × 10^6^ *P. brasiliensis* (Pb18) yeast cells, treated with an isotype control Ab or the anti-Ly6G specific mAb against neutrophils, and analyzed during the acute phase of* P. brasiliensis* infection (48 h and 96 h after challenge). Neutrophils were assessed by flow cytometry as described in the Materials and Methods section, which were identified as CD45^+^/CD11b^+^/Gr1^+^. (a) Representative flow cytometry contour plots of neutrophils within the gated CD45+ subpopulations are shown. Numbers inside the gates indicate the mean percentages of the gated subsets. (b) Bar plots of the neutrophils per lung. Data shown represent median and IQR (*n* = 4-5 mice/group; representative of two independent experiments). ^*∗*^ *P* < 0.05 comparing infected-untreated mice versus control mice or comparing infected-anti-Ly6G mAb-treated mice versus infected-untreated mice. (c) BALB/c mice were challenged intranasally with 1.5 × 10^6^ or 2 × 10^6^ *P. brasiliensis* yeast cells, treated as previously described, and evaluated over 12 weeks. Results are representative of two independent experiments (*n* = 10-11 mice/group). A statistically significant difference between the survival plots of the anti-Ly6G and the isotype or untreated mice was determined (*P* < 0.001). (d) Comparison of fungal burden in the lungs of BALB/c mice infected intranasally with 1.5 × 10^6^ *P. brasiliensis* yeast cells, treated or not as previously described, and sacrificed at 48 h and 96 h after challenge. Data shown represent median and IQR (*n* = 4-5 mice/group; representative of two independent experiments). A statistically significant increase in fungal burden was observed in the lungs of infected mice treated with the anti-Ly6G mAb (^*∗∗*^ *P* < 0.01) compared to the infected-untreated mice and the infected-isotype control Ab treated mice. PBS, control mice; PBS + Iso, control mice treated with isotype control Ab (clone: 2A3); PBS + antineutrophils, control mice treated with anti-Ly6G mAb (clone: 1A8); Pb18, infected-untreated mice; Pb18 + Iso, infected mice treated with isotype control Ab; Pb18 + antineutrophils, infected mice treated with anti-Ly6G mAb.

In order to investigate the efficiency of the mAb anti-Ly6G (clone 1A8) to deplete specifically neutrophils in BALB/c mice inoculated with PBS or 1.5 × 10^6^ *P. brasiliensis* yeast cells, a flow cytometry assay was carried out for lung homogenates at 48 h and 96 h after inoculation. The anti-Ly6G mAb depleted only neutrophils with an efficiency of 99.04% in* P. brasiliensis-*infected mice when compared to infected-untreated mice (Figure 1(a)). Untreated and isotype control-treated infected mice showed equivalent numbers of neutrophils, characteristic of this model at the early phase of infection (Figures 1(a) and 1(b)).

### 3.2. Neutrophils Are Essential to Control* P. brasiliensis* Infection during the Early Stages of PCM

To determine the role of neutrophils in the early stages of* P. brasiliensis* infection, we evaluated the survival time in untreated, isotype control, and mAb-treated mice after being infected with 1.5 × 10^6^ or 2 × 10^6^ *P. brasiliensis* yeast cells. We observed that 80% of neutrophil-depleted mice infected with 1.5 × 10^6^ yeast cells died within 2 weeks of infection; interestingly, when mice were infected with a slightly larger inoculum containing 2 × 10^6^ yeast cells, 100% of neutrophil-depleted animals succumbed by the first week postinfection, while most of the untreated or isotype-treated animals survived the 12-week assessment period (Figure 1(c)).

**Figure 2:**
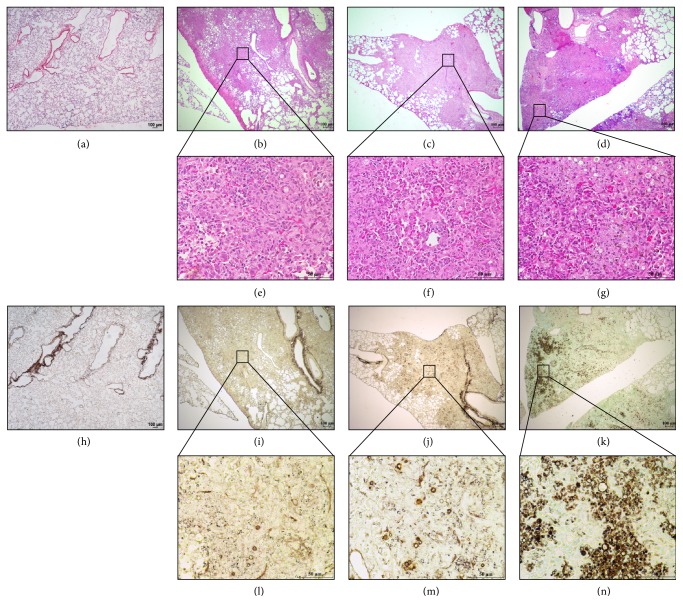
Depletion of neutrophils is associated with an exacerbated inflammatory response in lungs of mice infected with* P. brasiliensis* during the acute phase. The microphotographs shown are representative of lungs from mice infected or noninfected and treated or not with the anti-Ly6G specific mAb against neutrophils at 96 h after challenge and obtained from 4-5 mice/group. Lungs were fixed, embedded in paraffin, cut, and stained using H&E staining (a–g) to determine lung inflammatory response and methenamine silver staining (h–n) to identify* P. brasiliensis* yeast cells as described in the Materials and Methods section. Arrowheads indicate* P. brasiliensis* yeast cells (in brown). These results are representative of two independent experiments. (a) and (h) Control mice (inoculated with PBS); (b), (e), (i), and (l) infected, untreated mice; (c), (f), (j), and (m) infected mice treated with isotype control Ab; (d), (g), (k), and (n) infected mice treated with anti-Ly6G mAb. Magnification 40x (a–d, h–k) and 400x (e–g, l–o).

In order to investigate whether the decreased survival of infected and neutrophil-depleted mice was due to increased fungal load, we quantified fungal CFU obtained from lungs of mice at 48 and 96 h after infection with 1.5 × 10^6^ *P. brasiliensis* yeast cells. Neutrophil-depleted mice showed a significant increase in the pulmonary fungal burden compared to untreated and isotype control-treated infected mice (Figure 1(d)). These findings are consistent with the histopathological analysis which confirmed the increased pulmonary fungal burden as demonstrated by an increment of* P. brasiliensis* yeast cells in the lungs of neutrophil-depleted infected mice at the acute phase of infection (Figures 2(k) and 2(n)) compared to untreated and isotype control-treated infected mice (Figures 2(i), 2(j), 2(l), and 2(m)).

### 3.3. The Usage of the Specific mAb (Anti-Ly6G) Antineutrophils Did Not Affect Other Populations of Cells

**Figure 3:**
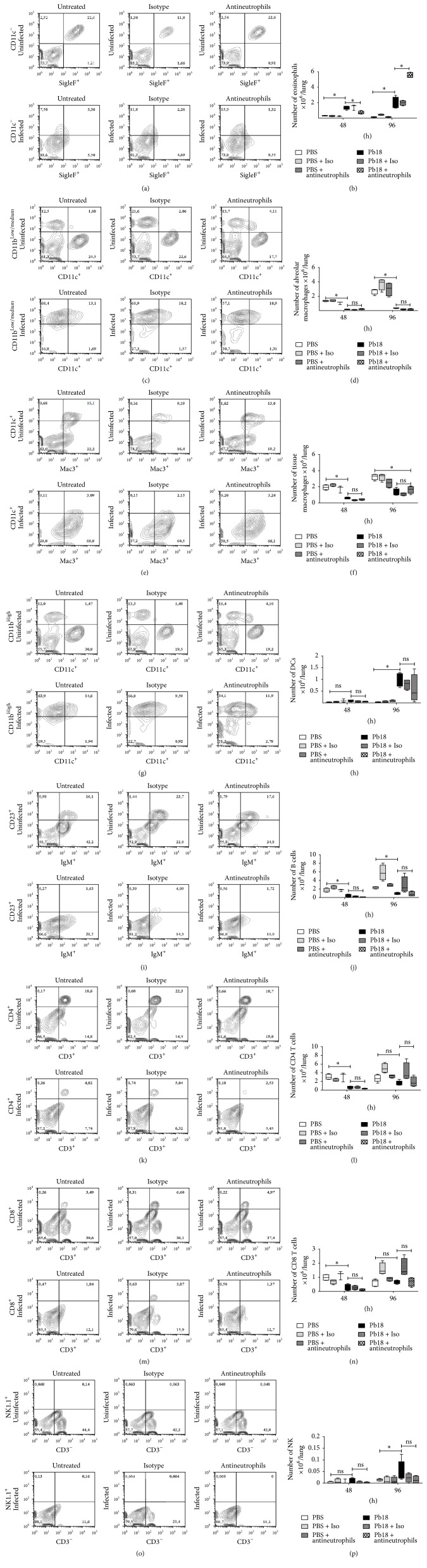
The anti-Ly6G mAb depletes specifically neutrophils and leads to an increased number of eosinophils during the acute stages of* P. brasiliensis* infection. BALB/c mice were intranasally inoculated with PBS or 1.5 × 10^6^ *P. brasiliensis* (Pb18) yeast cells, treated with an isotype control Ab or the anti-Ly6G specific mAb against neutrophils, and analyzed during the acute phase of* P. brasiliensis* infection (48 h and 96 h after challenge). Cell populations from lungs of mice were assessed by flow cytometry as described in the Materials and Methods section. Representative flow cytometry contour plots of pulmonary cells within the gated CD45+ subpopulations are shown as (a) eosinophils, (c) alveolar macrophages, (e), tissue macrophages, (g) DCs, (i) B cells, (k) CD4 T cells, (m) CD8 T, and (o) NK cells at 96 h after infection. Numbers inside the gates indicate the mean percentages of the gated subsets. Bar plots of the respective cell subpopulation per lung are shown (b, d, f, h, j, l, n, and p). Data shown represent median and IQR (*n* = 4-5 mice/group; representative of two independent experiments). ^*∗*^ *P* < 0.05 comparing infected-untreated mice versus control mice or comparing infected-anti-Ly6G mAb-treated mice versus infected-untreated mice. PBS, control mice; PBS + Iso, control mice treated with isotype control Ab (clone: 2A3); PBS + antineutrophils, control mice treated with anti-Ly6G mAb (clone: 1A8); Pb18, infected-untreated mice; Pb18 + Iso, infected mice treated with isotype control Ab; Pb18 + antineutrophils, infected mice treated with anti-Ly6G mAb; ns: not significant.

In order to evaluate the specificity of the anti-Ly6G mAb, we determined other cell populations by flow cytometry (eosinophils, alveolar and tissue macrophages, DCs, B cells, CD4 and CD8 T cells, and NK cells) during these early periods. We did not observe any difference in those cell populations among any of the groups of mice at 48 h or 96 h after inoculation with PBS or* P. brasiliensis* yeast (Figures 3(c)–3(p)), with the exception of eosinophils, which were significantly more abundant in the neutrophil-depleted infected mice group at 96 h postinfection (Figures 3(a) and 3(b)). Nonetheless,* P. brasiliensis* infection reduced the number of alveolar (Figures 3(c)-3(d)) and tissue (Figures 3(e)-3(f)) macrophages as well as B cells (Figures 3(i)-3(j)) in the all postinfection periods evaluated; CD4 (Figures 3(k)-3(l)) and CD8 T (Figures 3(m)-3(n)) cells were also diminished in number at 48 h after infection, while DC (Figures 3(g)-3(h)) and NK (Figures 3(o)-3(p)) cells were increased in number at 96 h after challenge in those infected animals.

In addition, histopathological analysis revealed an increased inflammatory reaction with a marked congestion, edema, and hemorrhage in neutrophil-depleted infected mice (Figures 2(d)–2(g)) compared with untreated or isotype-treated and infected mice at 96 h after infection (Figures 2(b), 2(c), 2(e), and 2(f)).

### 3.4. Depletion of Neutrophils in Mice Infected with* P. brasiliensis* Is Associated with Increased Levels of Chemokines and Proinflammatory Cytokines during the Early Phase of Infection

**Figure 4:**
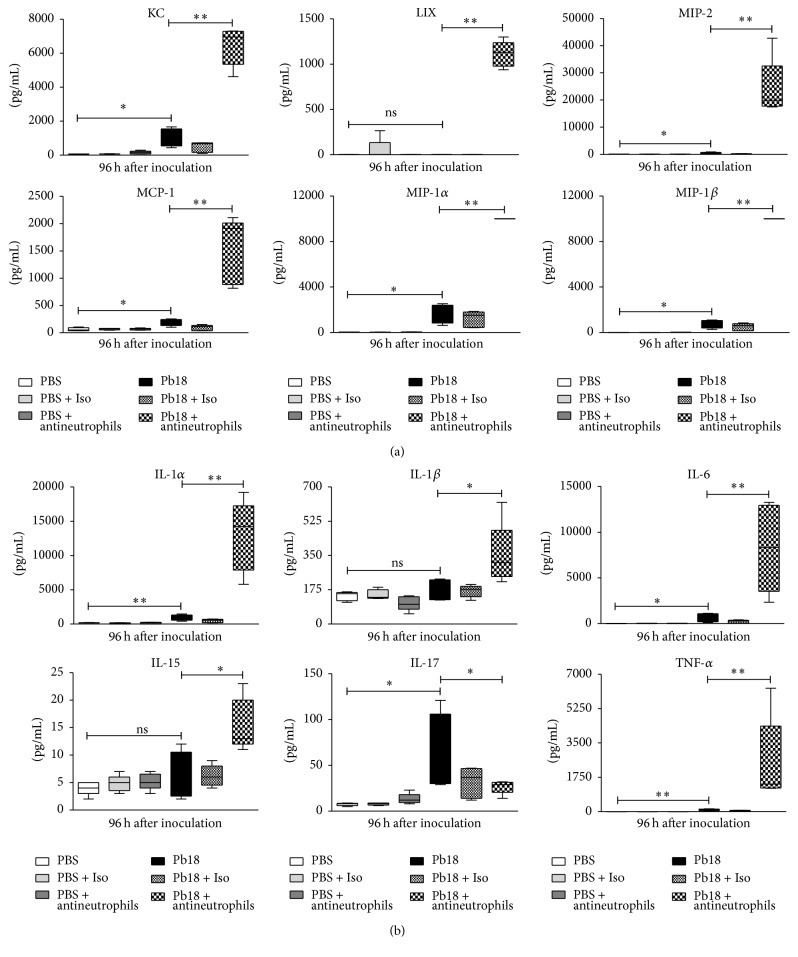
Depletion of neutrophils increases levels of chemokines and proinflammatory cytokines in lungs of mice infected with* P. brasiliensis* during the early stages of infection. BALB/c mice were challenged intranasally with 1.5 × 10^6^ *P. brasiliensis* (Pb18) yeast cells and treated with an isotype control Ab or the anti-Ly6G mAb specific to neutrophils. Supernatants from lung homogenates of mice sacrificed at 96 h after infection were analyzed using a commercial kit and the Luminex System as described in Material and Methods. (a) chemokines (KC, LIX, MIP-2, MCP-1, MIP-1*α*, and MIP-1*β*) and (b) proinflammatory cytokines (IL-1*α*, IL-1*β*, IL-6, IL-15, IL-17, and TNF-*α*). Data shown represent median and IQR (*n* = 4-5 mice/group; representative of two independent experiments). ^*∗*^ *P* < 0.05 and ^*∗∗*^ *P* < 0.01 comparing infected-untreated mice versus control mice or comparing infected-anti-Ly6G mAb-treated mice versus infected-untreated mice. PBS, control mice; PBS + Iso, control mice treated with isotype control Ab (clone: 2A3); PBS + antineutrophils, control mice treated with anti-Ly6G mAb (clone: 1A8); Pb18, infected-untreated mice; Pb18 + Iso, infected mice treated with isotype control Ab; Pb18 + antineutrophils, infected mice treated with anti-Ly6G mAb; ns: not significant.

**Figure 5:**
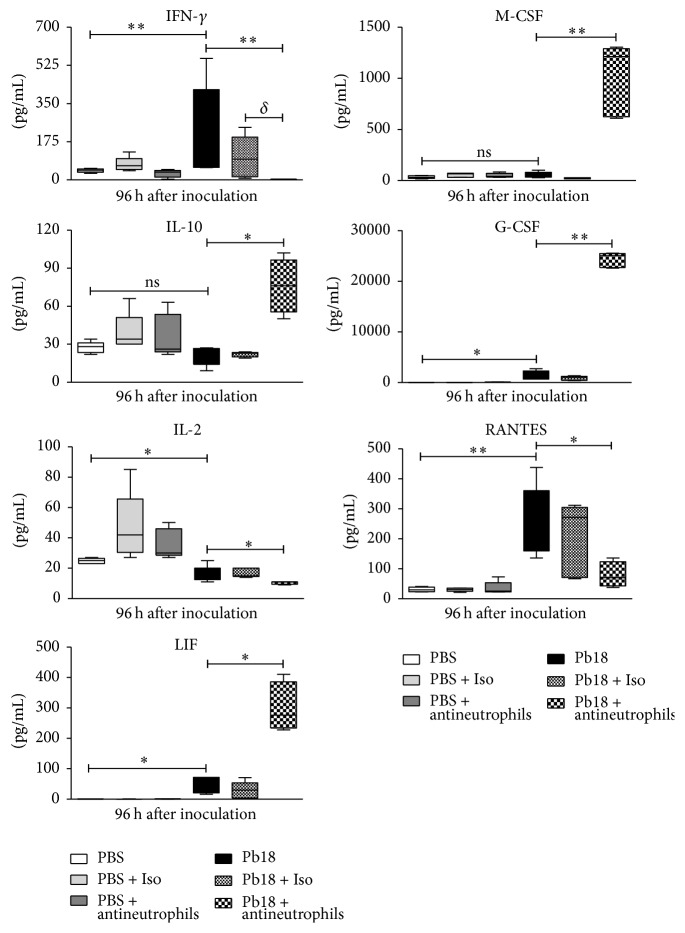
Depletion of neutrophils increases levels of growth factors, IL-10, and LIF but decreases IFN-*γ*, IL-2, and RANTES in lungs of mice infected with* P. brasiliensis* during the early stages of infection. BALB/c mice were challenged intranasally with 1.5 × 10^6^ *P. brasiliensis* (Pb18) yeast cells and treated with an isotype control Ab or the anti-Ly6G mAb specific to neutrophils. Supernatants from lung homogenates of mice sacrificed at 96 h after infection were analyzed using a commercial kit and the Luminex System as described in Material and Methods. Data shown represent median and IQR (*n* = 4-5 mice/group; representative of two independent experiments). ^*∗*^ *P* < 0.05 and ^*∗∗*^ *P* < 0.01 comparing infected-untreated mice versus control mice or comparing infected-anti-Ly6G mAb-treated mice versus infected-untreated mice. ^*δ*^ *P* < 0.01 comparing infected-isotype control antibody-treated mice versus infected-anti-Ly6G mAb-treated mice. PBS, control mice; PBS + Iso, control mice treated with isotype control Ab (clone: 2A3); PBS + antineutrophils, control mice treated with anti-Ly6G mAb (clone: 1A8); Pb18, infected-untreated mice; Pb18 + Iso, infected mice treated with isotype control Ab; Pb18 + antineutrophils, infected mice treated with anti-Ly6G mAb; ns: not significant.

We also quantified chemokine and proinflammatory cytokine levels in the lungs of infected and noninfected mice treated with the mAb and sacrificed at 48 and 96 h after inoculation. We observed that several chemokines and proinflammatory cytokines showed significantly higher levels in mice infected and treated with the specific mAb when compared with infected-untreated or isotype-treated mice. The molecules that depicted significant increases in those mAb treated-mice at 96 h after infection were CXCL1 (KC), CCL2 (MCP-1), CCL3 (MIP-1*α*), CCL4 (MIP-1*β*), CXCL2 (MIP-2), CXCL5 (LIX), IL-1*α*, IL-6, G-CSF, M-CSF, and TNF-*α* (for all, *P* < 0.01) and IL-1*β*, IL-15, IL-10, and LIF (for all, *P* < 0.05). In contrast, IFN-*γ* (*P* < 0.01), IL-2, IL-17, and CCL5 (RANTES) (for all, *P* < 0.05) were significantly lower (Figures 4 and 5). Similar results were observed at 48 h after infection (data not shown).

### 3.5. Neutrophils Are an Important Source of IFN-*γ* and IL-17

**Figure 6:**
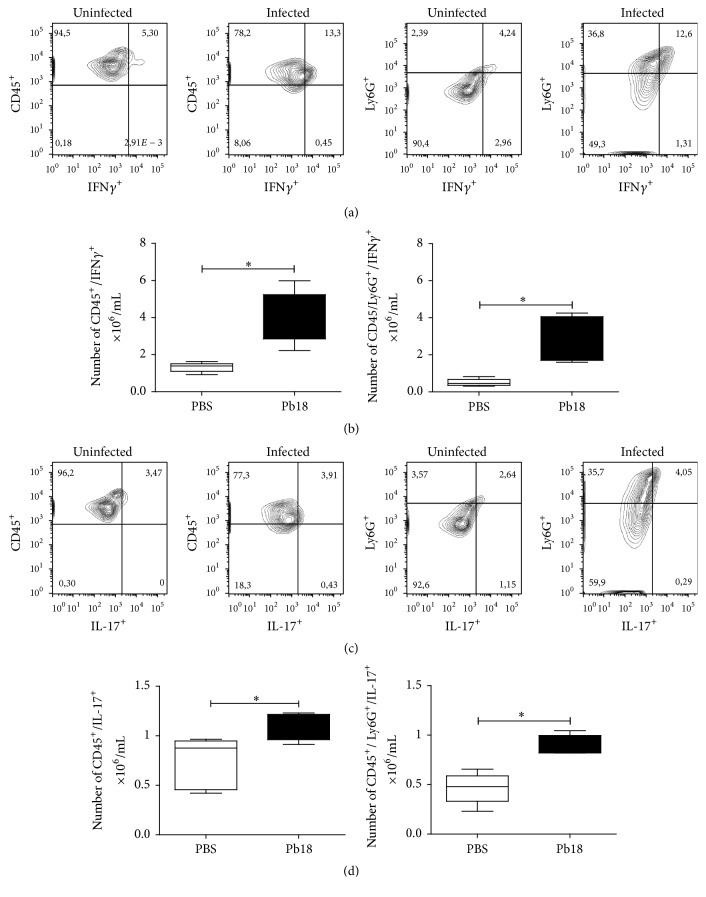
Neutrophils are an important source of IFN-*γ* and IL-17. BALB/c mice were intranasally inoculated with PBS or 1.5 × 10^6^ *P. brasiliensis* (Pb18) yeast cells and analyzed during the acute phase of* P. brasiliensis* infection (96 h postchallenge). (a) Representative flow cytometry contour plots of CD45^+^- and neutrophils- (CD45^+^/CD11b^+^/Gr1^+^/Ly6G^+^-) IFN-*γ*-producing cells subpopulations at 96 h after infection are shown. Numbers inside the gates indicate the mean percentages of the gated subsets. (b) Bar plots of CD45^+^- and neutrophils- (CD45^+^/CD11b^+^/Gr1^+^/Ly6G^+^-) IFN-*γ*-producing cells per lung. (c) Representative flow cytometry contour plots of CD45^+^- and neutrophils- (CD45^+^/CD11b^+^/Gr1^+^/Ly6G^+^-) IL-17-producing cells subpopulations at 96 h after infection are shown. Numbers inside the gates indicate the mean percentages of the gated subsets. (d) Bar plots of CD45^+^- and neutrophils- (CD45^+^/CD11b^+^/Gr1^+^/Ly6G^+^-) IL-17-producing cells per lung. Data shown represent median and IQR (*n* = 4-5 mice/group; representative of two independent experiments). ^*∗*^ *P* < 0.05 comparing infected versus control mice. PBS, control mice; Pb18, infected mice.

In other sets of experiments, we confirmed that neutrophils are a source of two important cytokines such as IFN-*γ*, which is necessary to activate macrophages and mount an effective antifungal response, and IL-17 considered one of the most important proinflammatory cytokines. Thus, near to 60% of the total leukocytes (CD45^+^)/IFN-*γ* producing cells corresponded to neutrophils (Figures 6(a)-6(b)). In the case of IL-17, neutrophils accounted for 73% of the total leukocytes (CD45^+^)/IL-17 producing cells (Figures 6(c)-6(d)).

## 4. Discussion

In the present study, we described neutrophils playing an important role during the initial immunopathology response in the acute stages of* P. brasiliensis* infection. Thus, during the early stages, depletion of these phagocytic cells in mice worsens the course of infection with an increase in both the fungal burden and the inflammatory response, causing a decrease in the survival rates. In addition, we described for the first time a murine model using the yeast cell morphotype to be inoculated by the intranasal route in order to develop the acute inflammatory stage as previously described in the animal model using the* P. brasiliensis* conidia morphotype [12].

In PCM, it has been demonstrated that neutrophils from patients with the disease have a significant deficiency in their ability to digest* in vitro* viable* P. brasiliensis* yeasts [31, 32]. Furthermore, enhanced antifungal activity of human neutrophils prior to activation with IFN-*γ*, GM-CSF, and/or IL-1*β* has been described, suggesting an important role of these phagocytic cells in host resistance during early infection with* P. brasiliensis* [33, 34]. Using animal models, a marked neutrophilic infiltration characteristic of the early cellular events in resistant (A/Sn) and susceptible (B10.A) mice after being intraperitoneally infected with* P. brasiliensis* has been demonstrated [35]. In another model, neutrophils obtained from subcutaneous air-pouches of resistant mice have shown a significantly increased production of hydrogen peroxide (H_2_O_2_) and high fungicidal activity at 15 days after infection, being more efficient in* P. brasiliensis* yeast cell killing compared to neutrophils from susceptible mice [26, 36]. Moreover, Gonzalez et al. [12] demonstrated a pulmonary infiltration of leukocytes with a predominance of neutrophils during the first 4 days after challenge with* P. brasiliensis* conidia in BALB/c mice, accompanied by elevated levels of proinflammatory cytokines such as TNF-*α*, IL-6, IL-1*β*, and CXCL2 (MIP-2). This suggests that both proinflammatory cytokine and leukocyte recruitment may influence the host immune response to control infection by* P. brasiliensis* conidia. Herein, we demonstrated that neutrophils are essential during the development of the innate immune response in BALB/c mice infected with* P. brasiliensis*; thus, their depletion led to an increase in fungal burden with subsequent early death.

Interaction of neutrophils with macrophages and DCs has been described [37]. In the case of macrophages, neutrophils are important contributors to their activation and recruitment at the site of infection through the release of cytokines and chemokines from activated neutrophils, such as IFN-*γ* that causes activation of macrophages [37, 38], while MIP-1*α*, MIP-1*β* [37, 39], and IL-17 [37, 40] attract them, suggesting that neutrophils may influence the phenomenon of macrophage differentiation into proinflammatory or anti-inflammatory subtype [37, 41, 42]. In turn, once macrophages are activated, neutrophil life span is prolonged at the site of inflammation by releasing IL-1*β*, TNF-*α*, G-CSF, and GM-CSF [37, 43, 44]. In the present study, we observed that* P. brasiliensis* infection decreased macrophages and in addition depletion of neutrophils, which is associated with low levels of IFN-*γ* and IL-17, could be responsible for the worsening or uncontrolled infection.

Regarding the neutrophil and DC interaction, neutrophils act as transport vehicle for intracellular pathogens delivering antigens to DCs suggesting an important role in the activation of T cell immune response by DCs [37, 45–47]. Upon activation, neutrophil granules release several inflammatory proteins with the potential to induce maturation of immature DCs [37, 48, 49]. Furthermore, the release of chemokines by neutrophils induces the attraction of immature DCs at the site of infection [37, 50]. In* Aspergillus fumigatus* infection, an immunomodulatory relationship was reported between neutrophils and DCs during the host response, promoting the maturation and efflux of lung DCs and suggesting that the reduced maturation of DCs in neutropenic hosts may inhibit the development and expansion of T cell-mediated immunity [51]. Although in this study we did not determine the maturation of DCs, reduction in maturation of these cells in neutropenic mice could not be ruled out, and thus the development of a specific cell-mediated immune response could be affected.

In recent years, several studies have elucidated a plethora of roles that neutrophils perform in a variety of diseases [2, 8, 52]. The importance of neutrophils in developing resistance against bacterial infection has recently been described through the usage of the mAb anti-Ly6G (1A8), indicating their essential role in recognizing and eliminating the bacteria as well as limiting their dissemination [53, 54]. In terms of fungal infections, the neutrophil has been considered to be an important determinant in the pathogenesis of invasive disease caused by* A. fumigatus* [23]. Mircescu et al. [55] subsequently demonstrated the essential role of neutrophils in early* A. fumigatus* infection using the mAb RB6-8C5, suggesting that these cells provide anticonidial defense by preventing germination and invasive disease. A study involving mice that were resistant (A/J) or susceptible (B10.A) to* P. brasiliensis* infection and using the anti-Gr1 mAb (RB6-8C5) to deplete neutrophils in order to determine their involvement of these cells in pulmonary PCM was done [26]; after depletion, mice were evaluated at 1 week after infection and mainly during the adaptive immune response development. In this study, the authors observed that susceptible mice began to die 161 days after infection, which was associated with increased pulmonary fungal load. In concurrence, in the present study, we observed that neutrophil-depleted animals died earlier (within two weeks) and showed higher fungal loads in their lungs, thus confirming that neutrophils are pivotal in the control of* P. brasiliensis* infection; nonetheless, it is important to note that we made some modifications to the animal model employed, such as (i) the specific mAb (anti-Ly6G), (ii) the intermediate susceptible mouse strain (BALB/c), (iii) the* P. brasiliensis* inoculum sizes (1.5 × 10^6^ and 2 × 10^6^ yeast cells), (iv) the mAb treatment schedule, and (v) the inoculation route (intranasal). These variables could account for the immune response developing in a different way from that observed in previously reported results. In addition, it is important to note that it has been reported that the RB6-8C5 mAb (anti-Gr1 or anti-Ly6C) depletes not only neutrophils but also monocytes, dendritic cells, and subpopulations of lymphocytes [19], a fact that clearly indicated that the neutrophils were not only the cells that could have the effect described previously. In the present work we confirmed that the mAb employed (anti-Ly6G) did not affect other cell populations different to neutrophils.

Interestingly, we observed a significant increase in levels of proinflammatory cytokines and chemokines besides eosinophils, as the only cell population depicting a significant change after depletion of neutrophils in infected mice. Similarly, Gonzalez et al. [56] observed that mice lacking NADPH oxidase (NOX2) activity (in which neutrophils are nonfunctional) and infected with* Coccidioides* presented elevated concentrations of proinflammatory cytokines, chemokines, and eosinophils in their lungs, a fact that was associated with an earlier death and higher fungal load. In the present study, we hypothesize that this dysregulation process could account for the worsening of the infection. Thus, elevated production of these molecules could be due to an attempt to recruit neutrophils at the infection site. On the other hand, the higher number of eosinophils observed in the lungs of neutrophil-depleted mice could be associated with the higher concentrations of CCL2 (MCP-1), CCL3 (MIP-1*α*), and CCL4 (MIP-1*β*) [57, 58] observed. Some authors have associated the prevalence of eosinophils with a Th2 pattern which is less efficient in controlling* P. brasiliensis* infection, as indicated by Mamoni et al. [59] who stated that eosinophilia and a Th2 pattern of cytokine expression, as well as IgE, IgG4, and IgA, are present in patients with the juvenile and multifocal adult forms of PCM. Similar findings have been described for coccidioidomycosis in which peripheral eosinophilia and eosinophilic microabscesses are correlated with progressive infection, indicative of a poor prognosis [59–61]. Additional studies support the hypothesis that the eosinophil participates in the pathophysiology of PCM through its toxic granule proteins [62–64]. In 2013, Muniz et al. [65] stated that the cytoplasmic granules of eosinophils are able to function as “cluster bombs” amplifying the differential secretory properties of these cells, releasing cationic proteins, cytokines, chemokines, and growth factors which could contribute to the persistence of infection, exacerbation of the inflammatory response, and subsequent death of* P. brasiliensis*-infected neutrophil-depleted mice observed in the present study.

It has been described that Th1-type immunity is characterized by activation of CD4+ T cells and secretion of IFN-*γ* and IL-12 which in turn stimulate a protective cell-mediated immune response to fungal infections, including* P. brasiliensis* [66, 67], while Th-17 pattern appears to be the major contributor to both the initiation and control of the inflammatory immune response to fungal infections [67]. Moreover, several studies indicate that IL-17 plays a key role in bridging between innate immunity and adaptive immunity [68]. Herein, we observed that cytokines, such as IFN-*γ* and IL-17, were significantly lower in neutrophil-deficient mice. Thus, it is important to note that although innate lymphocytes, mainly NK cells, are the main IFN-*γ* source during the innate immune response development [14, 69], in this study it was demonstrated that neutrophils are an important source of IFN-*γ* accounting for almost 60% of IFN-*γ*-producing cells from the total cell population in lungs. IFN-*γ* has been shown to be one of the most important cytokines able to activate macrophages, which in turn exert a potent antifungal effect against* P. brasiliensis* [57, 66, 70, 71]. Instead, during this early period, the anti-inflammatory cytokine IL-10 significantly increased in response to neutrophil depletion, possibly suggesting a negative modulation of the innate immune response against* P. brasiliensis* [15, 72]. In fact, IL-10 has been associated with the more severe forms of PCM [15, 73]. On the other hand, IL-17 is the characteristic cytokine produced by Th17 cells, which together with IL-22 activates mononuclear phagocytes, recruits neutrophils, and induces epithelial antimicrobial response, protecting against extracellular bacteria and fungi [74]. Although IL-17 has been mainly associated with autoimmune or chronic infectious diseases [37, 75], it plays a significant role as first line of defense against infections during the innate immune response [37, 76, 77]. Herein, the significant decrease of IL-17 in neutrophils-depleted and infected mice suggested that neutrophils may be important contributors to the production of IL-17 at the onset of PCM, which was demonstrated by intracellular flow cytometry showing that neutrophils represented almost 73% of IL-17-producing cells from the total cell population in lungs. These findings prompted us to hypothesize that the absence of neutrophils associated with the lack of important cytokines during the innate immune response, such as IFN-*γ* and IL-17, could be, in part, responsible for the progression of an uncontrolled* P. brasiliensis* infection. Thus, Th1- and Th17-type immunity could share specific tasks in the control of fungal infections [67].

Finally, we observed increased levels of several proinflammatory cytokines and chemokines (Figure 4) in neutrophils-depleted infected mice; all of them accomplish neutrophils chemoattractant functions [57, 58, 78, 79]. Thus, the increased levels of such molecules could be explained by accumulation due to the absence of neutrophils in mAb-treated mice and possibly by the low levels of IFN-*γ* that is able to downregulate their expression [57, 80].

## 5. Conclusions

The results of the present study indicate that, besides the microbicidal effects, neutrophils exert an immunomodulatory effect during the innate immune response in experimental PCM. Our findings demonstrated that neutrophils are able to produce IFN-*γ* and IL-17 cytokines, which have important effects on macrophages activation enabling them to exert an effective antifungal activity against* P. brasiliensis*. Thus, depletion of neutrophils and the subsequent decrease of IFN-*γ* and IL-17 levels caused an uncontrolled immune response characterized by increased fungal load in lungs, a fact that reflects that the deficiency to eliminate the fungus may be due to lack of macrophages and DCs activation, accumulation of eosinophils, and increased levels of proinflammatory cytokines and chemokines that triggered the early death of the host. Finally, these findings may be supporting the hypothesis that IFN-*γ* and IL-17 produced by neutrophils, among other factors, could address the immune response bridging innate and adaptive immunity in* P. brasiliensis* infection [38].
